# Zinc Maintains Embryonic Stem Cell Pluripotency and Multilineage Differentiation Potential via AKT Activation

**DOI:** 10.3389/fcell.2019.00180

**Published:** 2019-08-30

**Authors:** Hayk Mnatsakanyan, Roser Sabater i Serra, Manuel Salmeron-Sanchez, Patricia Rico

**Affiliations:** ^1^Centre for Biomaterials and Tissue Engineering (CBIT), Universitat Politècnica de València, Valencia, Spain; ^2^Biomedical Research Networking Centre in Bioengineering, Biomaterials and Nanomedicine (CIBER-BBN), Madrid, Spain; ^3^Division of Biomedical Engineering, Centre for the Cellular Microenvironment, School of Engineering, University of Glasgow, Glasgow, United Kingdom

**Keywords:** zinc, ZIP7, stemness maintenance, embryonic stem cells (ESC), AKT

## Abstract

Embryonic stem cells (ESCs) possess remarkable abilities, as they can differentiate into all cell types (pluripotency) and be self-renewing, giving rise to two identical cells. These characteristics make ESCs a powerful research tool in fundamental embryogenesis as well as candidates for use in regenerative medicine. Significant efforts have been devoted to developing protocols to control ESC fate, including soluble and complex cocktails of growth factors and small molecules seeking to activate/inhibit key signaling pathways for the maintenance of pluripotency states or activate differentiation. Here we describe a novel method for the effective maintenance of mouse ESCs, avoiding the supplementation of complex inhibitory cocktails or cytokines, e.g., LIF. We show that the addition of zinc to ESC cultures leads to a stable pluripotent state that shares biochemical, transcriptional and karyotypic features with the classical LIF treatment. We demonstrate for the first time that ESCs maintained in long-term cultures with added zinc, are capable of sustaining a stable ESCs pluripotent phenotype, as well as differentiating efficiently upon external stimulation. We show that zinc promotes long-term ESC self-renewal (>30 days) via activation of ZIP7 and AKT signaling pathways. Furthermore, the combination of zinc with LIF results in a synergistic effect that enhances LIF effects, increases AKT and STAT3 activity, promotes the expression of pluripotency regulators and avoids the expression of differentiation markers.

## Introduction

Embryonic stem cells (ESCs) are the main cellular source during embryogenesis in multicellular organisms, giving rise to the three embryonic germ layers (mesoderm, endoderm and ectoderm) which are the precursors required for the development of functional tissues (Niwa, 2007; Ohtsuka and Dalton, 2008; Evans, 2011). ESCs present a pluripotent phenotype, being able to self-renew and propagate indefinitely in appropriate conditions or differentiate into all embryonic cell lineages spontaneously (Tremml et al., 2008) or in a controlled way (Wichterle et al., 2002; West et al., 2006; Schugar et al., 2008; Evseenko et al., 2010; Kattman et al., 2011). This multilineage differentiation potential, makes them a key tool in research on biological processes, developing new prospects for the medical treatment of different pathologies and as a cell source for regenerative medicine strategies (Clevers, 2016; Yiangou et al., 2018).

ESCs can be obtained from the inner cell mass of the preimplantation blastocyst stage (Tremml et al., 2008; Czechanski et al., 2014). *In vitro* ESC expansion is challenging due to its marked tendency to spontaneously differentiate into all primary germ layers (Heo et al., 2005; Nair et al., 2015). They thus need to be cultured in very specific conditions that mimic the *in vivo* niche to inhibit the activation of differentiation mechanisms and promote self-renewal (Stewart et al., 2008; Llames et al., 2015). The most common method of maintaining undifferentiated ESC phenotypes *in vitro* is their co-culture onto a feeder layer of inactivated mouse embryonic fibroblasts (MEF) (Tamm et al., 2013; Llames et al., 2015), which provides ESC paracrine factors for stemness maintenance (Llames et al., 2015). However, these feeder cells exhibit a heterogeneous population with different surface markers and phenotypes (Singhal et al., 2016) which entail a source of variability during ESC culture.

Among the pool of molecules released by the feeder layer of cells, some growth factors and cytokines involved in the inhibition of ESC differentiation have been identified, such as the Leukemia inhibitory factor (LIF) (Llames et al., 2015). LIF binds to the glycoprotein 130/LIF-receptor and activates multiple signaling pathways, including the tyrosine kinase Janus (JAK) (Ohtsuka and Dalton, 2008; Pera and Tam, 2010; Oshimori and Fuchs, 2012). When JAK phosphorylates, it downstream activates both the PI3K/AKT cascade and the transcription factor STAT3, regulating the expression of self-renewal associated genes (Niwa et al., 1998). Other factors identified are the fibroblast growth factor-2 (FGF2), Activin A, Gremlin 1 or transforming growth factor β1 (TGFβ1), which inhibit human ESC differentiation (Stewart et al., 2008). It is, however, often necessary to combine more than one molecule to achieve strong inhibition of ESC differentiation (Stewart et al., 2008), particularly for human ESCs. Besides the cell-released factors, there is a growing trend toward the study of small molecules to drive ESC fate (Li and Ding, 2010). Among these small molecules, it has been shown that GSK3β inhibitors (such as CHIR 99021; Bechard and Dalton, 2009; Tamm et al., 2013) or ERK1/2 inhibitors (such as SC1, Chen et al., 2006 or PD184352, Kunath et al., 2007; Hamilton and Brickman, 2014), directly or indirectly target the POU5F1, SOX2, NANOG, and KLF4 (Jaenisch and Young, 2008; Li and Ding, 2010), group of transcription factors or protein kinases, such as PI3K/AKT, which form pluripotency associated regulators (Yu and Cui, 2016).

PI3K/AKT signaling is crucial to promote ESC survival by inhibiting apoptosis through phosphorylation and inactivation of BAD or Caspase-9 (Kennedy et al., 1997; Cardone, 1998). In addition, AKT is able to downstream regulate the activity of protein kinases and transcription factors such as GSK3β, NFκβ, MEK/ERK (Armstrong et al., 2006; Yu and Cui, 2016), or POU5F1 (Lin et al., 2012), which control the activity and expression of proliferation and pluripotency-associated genes. GSK3β and ERK activity are commonly associated with loss of pluripotency. GSK3β binds and phosphorylates β-Catenin, triggering its proteasomal degradation (Doble, 2003), and consequently disrupts the Wnt/β-Catenin signaling, necessary for preserving ESC integrity and somatic cell reprogramming (Berge et al., 2011; Xu et al., 2016). In the same way, ERK activity is closely related to the suppression of ESC self-renewal and specification to endoderm lineage (Hamilton and Brickman, 2014). Inhibition of both protein kinases (GSK3β and ERK), known as the 2i condition, enhances ESC self-renewal, avoiding differentiation (Morgani et al., 2013). On the other hand, NFκβ and POU5F1 transcriptional activity are absolutely necessary for pluripotency gene expression (Armstrong et al., 2006). The role of the PI3K/AKT pathway in the regulatory process of ESC survival and pluripotency makes it an interesting target to promote ESC self-renewal. Among the molecules that activate the PI3K/AKT cascade, growth factors such as FGF2 (Johnson-Farley et al., 2007; Eiselleova et al., 2009), IGF1/2, Insulin (Johnson-Farley et al., 2007; Franke, 2008) or small molecules such as SC79 (Jo et al., 2012) or zinc have been reported (Taylor et al., 2008).

Zinc is a transition metal involved in the activity of several proteins, such as transcription factors or enzymes, and may act as a neurotransmitter (Murakami and Hirano, 2008). In addition, the role of Zn^2+^ has been established as an intracellular second messenger down regulating phosphatase activity (Yamasaki et al., 2007), which upgrades the phosphorylation of several protein kinases such as ERK, JNK, or AKT (Yamasaki et al., 2007; Taylor et al., 2012), promoting their activity. Several authors have reported that zinc ions are involved in the activation of the PI3K/AKT cascade through phosphorylation of AKT (Taylor et al., 2012; Cohen et al., 2014; Shao et al., 2017). After addition of extracellular concentrations of zinc, intracellular Zn^2+^ concentration increases, a phenomenon that is controlled by the ZIP7 transporter in the endoplasmic reticulum (Yamasaki et al., 2007; Taylor et al., 2008, 2012). Subsequently ZIP7 inhibits phosphatase activity and triggers Zn-associated AKT phosphorylation, similarly to growth factor stimuli (Taylor et al., 2012). The role of Zn^2+^ in the transient inhibition of murine ESC differentiation has only recently been described, associating this behavior with the activation of the STAT3 transcription factor (Hu et al., 2016). However, it is noteworthy that STAT3 activity is regulated by the protein EZH2, which in fact is a substrate for AKT-mediated phosphorylation (Zhou et al., 2007; Kim et al., 2013), opening up the possibility of STAT3 activity being a consequence of upstream activation of the PI3K/AKT pathway.

Here we unravel the mechanism by which zinc ions controlled ESCs and set up new strategies to control the pluripotency of ESCs. We demonstrate the role of extracellular Zn^2+^ on the fate of mouse ESC through the PI3K/AKT signaling cascade. We show that the addition of Zn^2+^ only, induces self-renewal mechanisms and maintains pluripotency after long-term cultures. We also show that Zn^2+^ successfully reinforces the effect of LIF, and avoids spontaneous ESC differentiation. We thus have identified zinc as an alternative to the various and complex cocktails of small molecules and growth factors classically used for stable ESC maintenance *in vitro*.

## Materials and Methods

### ESCs Culture and Embryoid Body Formation

Murine embryonic stem cells D3 (ESCs, ATCC) were cultured in feeder-free conditions in basal medium (BM) composed of DMEM high glucose (Lonza) supplemented with 10% Knockout Serum Replacement (KSR, Thermo Fisher Scientific), 1% Fetal Bovine Serum (FBS, Gibco), 1% 100X Nucleosides (Millipore), 1% L-Glutamine (Sigma-Aldrich), 1% Non-essential Amino Acids (Sigma-Aldrich), 1% Penicillin/Streptomycin (P/S, Gibco) and 10 mM 2-Mercatoethanol (Gibco) at 37°C in a humidified incubator containing 5% CO_2_. 1,000 U/ml Leukemia inhibitory factor (LIF, Millipore) was used to inhibit ESC differentiation (growth medium). Before seeding, culture dishes were coated with 0.2% gelatin (Sigma-Aldrich). Zinc chloride (Sigma-Aldrich) was used as source of Zn^2+^ for *in vitro* experiments.

For embryoid bodies (EBs), ESCs previously grown for 30 days (30d-ESC) in BM (30d-BM) BM supplemented with Zn 100 μM (30d-Zn) or LIF (30d-LIF) were cultured in hanging drops (1,000 cells/20 μL drop). EBs for histological cuts were collected after 5 days of culture, transferred to non-adhesive flasks, and cultured for 10 additional days prior to histological analysis. EBs used for spontaneous ESC differentiation were cultured in DMEM/F12 medium (Sigma-Aldrich) supplemented with 10% FBS, 1% L-Glutamine, 1% P/S.

### ESCs Intracellular Zn^2+^ Homeostasis

For analysis of intracellular Zn^2+^ wave induction, cells were incubated with FluoZin3-AM (2 μM) for 40 min and washed with PBS, before adding medium at different zinc concentrations (40, 100, and 140 μM). Fluorescence emission was measured every 40 s for approximately 60 min. BM and BM supplemented with LIF were used as control. Fluorescence emission was analyzed by a Victor III plate reader (Perkin Elmer).

### Immunofluorescence, Staining, and Histology Methods

For immunofluorescence protein detection, cells were fixed with 4% formaldehyde for 20 min at room temperature. Cells were then washed with TBS and blocked with TBS/Triton × 100 0.1%/BSA 2% for 1 h at room temperature and incubated with primary antibody overnight at 4°C (Supplementary Table 1). The samples were subsequently washed and incubated with secondary antibodies for 1 h at room temperature (Supplementary Table 1). Hoechst (dil: 1/7,000, Sigma-Aldrich) was used for nuclear staining. Samples were mounted with 85% glycerol and imaged by a Nikon Eclipse i80 fluorescence microscope. The percentage of protein staining was quantified by image analysis with imageJ software.

To analyze the number of alkaline phosphatase-positive (AP) colonies, ESCs were fixed with 4% formaldehyde and stained with Naphthol AS-MX/Fast Red TR (Sigma-Aldrich) for 30 min at room temperature, as previously described (Ziomek et al., 1990). After staining, cells were rinsed and nuclei were labeled with Hoechst. The samples were imaged by a Nikon Eclipse i80 fluorescence microscope. The percentage of AP stained colonies was evaluated by imageJ software.

For histological sections, EBs were fixed with 4% formaldehyde overnight at 4°C. EBs were embedded into low gelling temperature agarose (Sigma-Aldrich) and paraffin (Polyester Wax, Electron Microscopy) for 6 μm histological sections obtained with HM 350 S (Thermo Fisher Scientific). Paraffin sections were histochemically stained with Haematoxylin/Eosin (Sigma-Aldrich), as previously described (Fischer et al., 2008).

### RNA Interference (RNAi)

ESCs were seeded at high density (40,000 cells/cm^2^) in growth medium (+ LIF). After 24 h, cells were transfected with MISSION esiRNA (Sigma-Aldrich) against ZIP7 (*Slc39a7*) transporter in X-tremeGENE siRNA Transfection Reagent (Roche), following the manufacturer’s instructions. Cell transfection was carried out in Opti-MEM Reduced Serum medium (Thermo Fisher Scientific) supplemented with LIF. MISSION siRNA Fluorescent Universal Negative Control 1, Cyanine 3 (NC, Sigma-Aldrich) was used as negative control. Transfected ESCs were cultured for 24 h, after which transfection medium was changed for basal medium supplemented with 100 μM Zn^2+^, 1,000 U/mL LIF or BM only and cultured for an additional 7 days.

### AKT Inhibition Experiments

For PI3K/AKT specific inhibition, we used 2-(4-Morpholinyl)-8-phenyl-1(4H)-benzopyran-4-one hydrochloride (LY-294002, Sigma-Aldrich), at a concentration of 10 μM. ESCs were seeded at 20,000 cells/cm^2^ in growth medium. After 24 h, growth medium was replaced for BM supplemented with 100 μM Zn^2+^, LIF or BM only and LY-294002. After 2 days, LY-294002 depleted medium was replaced in the different conditions for 7 days.

### Western Blot Analysis

For protein expression analysis, total protein extraction was performed with RIPA buffer supplemented with protease inhibitor cocktail tablets (Roche). Proteins were separated in 10% SDS-PAGE with Mini-PROTEAN Electrophoresis System (Bio-Rad) and transferred to a PVDF (GE-Healthcare) membrane by Trans-Blot Semi-Dry Transfer Cell (Bio-Rad). Membranes were then blocked with 5% BSA (Sigma-Aldrich) in TBS/0.1% Tween 20 (Sigma-Aldrich). For subsequent incubation with primary antibodies (Supplementary Table 2) they were diluted on TBS 0.1% Tween 20 and 3% BSA and incubated overnight at 4°C. Membranes were washed and incubated with HRP-linked secondary antibody (Supplementary Table 2) for 1 h at room temperature for chemiluminiscence band detection by ECL-Plus reactive (Thermo Fisher Scientific). A Fujifilm Las-3000 imager device was used for protein band visualization.

For detection of phosphorylated proteins (pAKT and pGSK3β), PVDF membranes were first incubated with specific antibodies against phosphorylated forms of the proteins (pAKT, pGSK3β) following the standard procedure explained before. After that, membranes were stripped using a detergent-containing buffer Western Blot Stripping Buffer (Thermo Fisher Scientific) for anti-phospho antibodies removal, and afterward reprobed again with the antibodies against the total protein (non-phosphorylated AKT and GSK3β) as a loading control.

### Gene Expression Analysis by Quantitative Real Time PCR

Total RNA was extracted from ESCs using Quick RNA Miniprep kit (ZYMO Research); quantity and integrity were measured on a Q3000 micro volume spectrophotometer (Quawell). RNAs were reverse transcribed by a Maxima First Strand cDNA Synthesis Kit with Thermolabile dsDNase (Thermo Fisher Scientific). Real-time qPCR was carried out on a PowerUp SYBR Master Mix (Thermo Fisher Scientific) and ViiA 7 Real-Time PCR System (Thermo Fisher Scientific). The reactions were run four times (independent biological experiments). The primers used for amplification are indicated in Supplementary Table 3. The fractional cycle number at which fluorescence passed the threshold (C_t_ values) was used for gene expression quantification by the comparative ΔΔC_t_ method. Sample values were normalized to the threshold value of *Gapdh* housekeeping gene.

### Statistical Analyses

Each experiment was performed at least four times unless otherwise noted. Data were reported as mean ± standard deviation. The D’Agostino-Pearson omnibus test was used to determine whether the data obtained followed a normal distribution. Results were analyzed by one-way ANOVA on GraphPad Prism 6.0. When differences were found to be significant, Tukey pairwise comparisons were performed on normal data distributions or a Dunn’s test in the opposite case. A 95% confidence level was considered significant. The linear regression of immunofluorescence image quantification and Pearson’s r correlation values was obtained on R software.

## Results

### Zn^2+^ Influences ESC Viability and Proliferation

To determine zinc concentrations that are toxic for ESCs, cell viability was analyzed using concentrations of Zn^2+^ from 40 to 240 μM at 1, 4, and 7 days (Supplementary Figure 1). ESC viability was maintained with Zn^2+^ concentrations of up to 140 μM, and decreased significantly at concentrations of 160 and 240 μM (Supplementary Figure 1A).

For proliferation experiments, Zn^2+^ concentrations that compromised ESC viability (over 160 μM) were discarded. Total DNA was measured after supplementing cells with 40, 100, and 140 μM Zn^2+^ for 1, 3, 5 or 7 days. In Supplementary Figure 1B no differences can be seen in cell proliferation for the different Zn^2+^ concentrations, which remained similar to those of the LIF and basal medium conditions.

### Zn^2+^ Promotes ESCs Self-Renewal

To study the mechanisms induced by soluble Zn^2+^ in ESCs, we first measured the Zn^2+^ cytosolic intake. The intracellular Zn^2+^ concentration was quantified for different concentrations of supplemented extracellular Zn^2+^ after 1, 3, and 5 days of culture. Free intracellular Zn^2+^ was labeled with FluoZin3-AM (Supplementary Figure 1C). Intracellular Zn^2+^ rose monotonically with extracellular Zn^2+^ concentration. The culture was maintained for 5 days, after which Zn^2+^ was removed from the medium and the culture left for 1 h, which led to a drastic drop in intracellular Zn^2+^ concentration [reaching levels similar to the basal medium (BM)]. This demonstrates that intracellular ESC zinc concentration is regulated by the concentration of extracellular Zn^2+^ in the culture medium.

**FIGURE 1:**
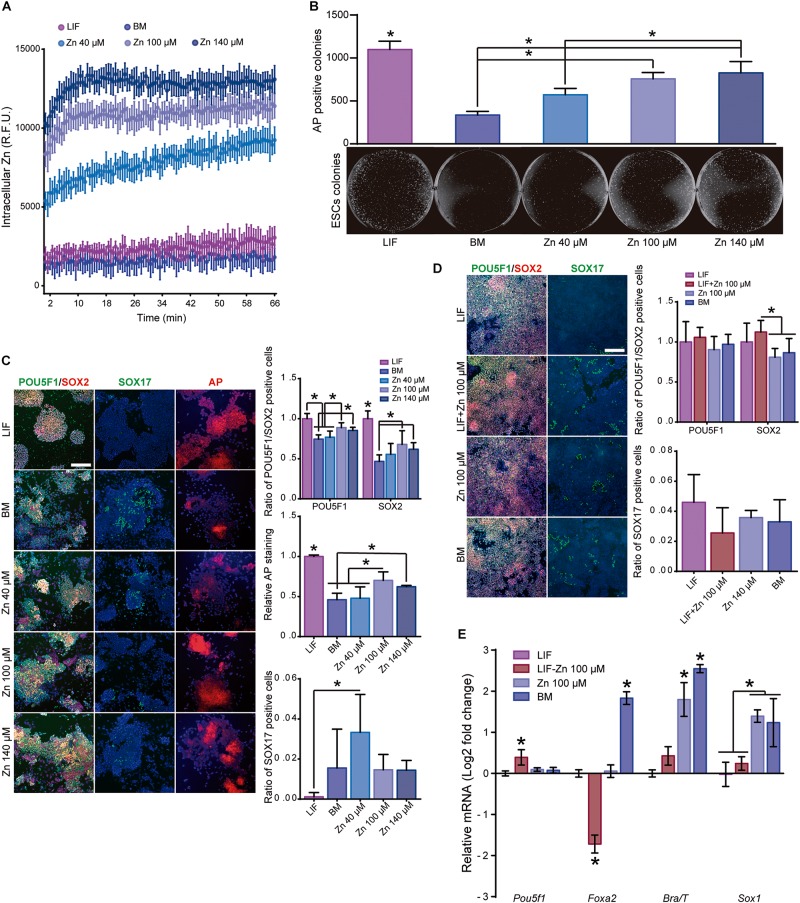
Role of zinc in ESC self-renewal. **(A)** Intracellular Zn^2+^ wave determined by FluoZin3-AM labeling (data points were taken every 40 s). Cells were kept at 37°C throughout the analysis (*n* = 6). **(B)** AP staining of ESCs after 5 days of culture in BM, medium supplemented with LIF or medium supplemented with different concentrations of Zn^2+^ (*n* = 3). **(C)** Immunofluorescence detection of pluripotency markers (POU5F1 and SOX2) and staining of AP in ESCs after 5 days of culture in BM, medium supplemented with LIF or medium supplemented with different concentrations of Zn^2+^ (*n* = 5). **(D)** Immunofluorescence detection of pluripotency (POU5F1 and SOX2) and differentiation markers (endoderm: SOX17) determined after 5 days of culture in accelerated differentiation conditions (medium supplemented with 5% KSR) (*n* = 5). **(E)** qPCR detection of pluripotency markers (*Pou5f1*) and differentiation markers (endoderm: *Foxa2*, mesoderm: *Brachyury/T* and ectoderm: *Sox1*) determined after 5 days of culture in accelerated differentiation conditions (medium supplemented with 5% KSR). *Gapdh* was used as housekeeping gene (*n* = 4). Significant differences were determined by ANOVA test; ^∗^*p* < 0.05. Scale bar: 200 μm.

As Zn^2+^ is an intracellular secondary messenger involved in multiple cellular events (Yamasaki et al., 2007), we then measured intracellular “zinc waves” in ESCs after adding Zn^2+^ to the culture medium. Figure 1A shows that intracellular Zn^2+^ rose rapidly on the addition of 100 and 140 μM and reached a plateau after 10 min. However, supplementing with 40 μM Zn^2+^ led to a steady uptake of intracellular Zn^2+^, which was still increasing after 60 min. Intracellular Zn^2+^ did not rise in the basal medium (BM) and LIF supplemented medium (LIF) used as controls.

We next assessed the effect of Zn^2+^ on ESC self-renewal using basal medium (BM) and media supplemented with 40, 100, or 140 μM Zn^2+^ (Figures 1B,C). LIF-supplemented medium was used as a positive control for stemness maintenance. To determine the potential of ESCs to form alkaline phosphatase (AP) positive colonies in the presence of Zn^2+^, cells were seeded at 150 cells/cm^2^ and cultured for 5 days. The quantification of the AP expressing colonies indicated that the number of ESCs colonies increased monotonically as the extracellular concentration of Zn^2+^ does (Figure 1B). Then, ESCs were seeded at low density (10,000 cells/cm^2^) until sub-confluence (3 days) and subsequently sub-cultured for a total of 6 days. We used POU5F1, SOX2, and AP as pluripotency markers, and SOX17 as spontaneous differentiation marker. Figure 1C shows that only 100 and 140 μM Zn^2+^ concentrations produced high levels of POU5F1 and SOX2 expression as well as larger AP positive colonies than in the ESCs cultured in BM. Using 40 μM Zn^2+^-treated cultured cells was not enough to maintain pluripotency markers that remain similar to the BM control. Accordingly, 40 μM Zn^2+^-treated ESCs expressed the highest SOX17 values. These results show that adding sufficiently high concentrations of Zn^2+^ to the culture medium maintains ESCs stemness for more than 6 days.

To show that Zn^2+^ promotes ESC self-renewal, we performed additional experiments to induce cell differentiation using defined media in the presence of Zn^2+^. ESCs were seeded at 10,000 cells/cm^2^ in BM supplemented with LIF. After 24 h of culture, the medium was replaced by BM supplemented with 5% KSR (to stimulate ESC differentiation; Hamatake et al., 2000) and 100 μM Zn^2+^, LIF, or a combination of both LIF and 100 μM Zn^2+^. Figures 1D,E show immunofluorescence and qPCR pluripotency expression values (POU5F1 and SOX2) and primary germ layer differentiation markers (endoderm: *Foxa2*; mesoderm: *Brachyury/T*; ectoderm: *Sox1*) after 5 days of culture. ESCs treated with either LIF or Zn^2+^ had lower expressions of differentiation markers than the BM control. Endoderm marker expression (*Foxa2* and *Sox17*) were similar in the Zn^2+^ and LIF-treated cells. However, Zn^2+^-treated ESCs showed an upregulated expression of ectoderm and mesoderm markers (*Brachyury/T* and *Sox1*). However, the best results in terms of pluripotency were obtained with a combination of LIF and 100 μM Zn^2+^. In addition to downregulating the expression of differentiation markers, ESCs treated with LIF-100 and μM Zn^2+^ also displayed a slight overexpression of *Pou5f1*. These results further demonstrate that zinc maintains ESC self-renewal potential even under accelerated differentiation conditions.

### Zn^2+^ Maintains ESCs Stemness Activating ZIP7 Transporter and AKT Signaling Transduction Pathway

Zn^2+^ transporter ZIP7 is found in different organelles and cytoplasmic vesicles associated with Zn^2+^ storage (Huang et al., 2005; Hogstrand et al., 2009), and drives Zn^2+^ influx from intracellular storages to cytoplasm. ZIP7 activation has been extensively associated with AKT phosphorylation (Taylor et al., 2008), which is a key protein kinase regulator of the ESC self-renewal pathway (Watanabe et al., 2006). To determine the role of ZIP7 and AKT in ESCs we designed two experiments to evaluate the effect of zinc: (i) AKT inhibition with the PI3K/AKT chemical inhibitor LY-294002, and (ii) transient down regulation of ZIP7 transporter using siRNAs.

**FIGURE 2:**
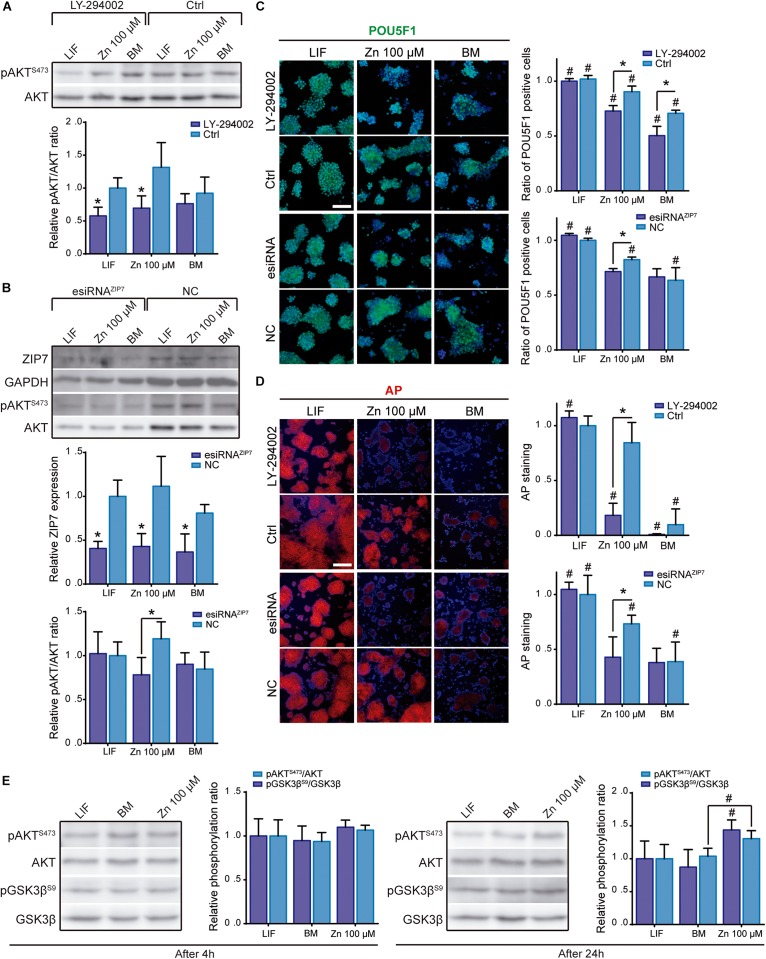
Role of ZIP7 and AKT in ESCs stemness maintenance. **(A)** Analysis of pAKT/AKT ratio by western blot after AKT inhibition with LY-294002 10 μM during 24 h in cells cultured in BM, medium supplemented with LIF or medium supplemented with 100 μM of Zn^2+^. GAPDH was used as loading control protein (*n* = 4). **(B)** Analysis of ZIP7 expression and pAKT/AKT ratio by western blot after ZIP7 silencing with RNAi after 3 days of culture. GAPDH was used as loading control protein (*n* = 4). Cells were cultured in BM and a medium supplemented with LIF or medium supplemented with 100 μM of Zn^2+^. **(C,D)** Immunofluorescence detection of pluripotency markers (POU5F1-green and alkaline phosphatase-red, AP) determined after 7 days of culture in BM, medium supplemented with LIF or medium supplemented with 100 μM of Zn^2+^, and treated 24 h with AKT inhibitor LY-294002, or ZIP7 silenced with RNAi during 3 days. Scale bar: 100 μm. **(E)** Analysis of pAKT/AKT (S473) and pGSK3β/GSK3β (S9) ratios by western blot in ESCs cultured in BM, medium supplemented with LIF or medium supplemented with 100 μM of Zn^2+^ for 4 or 24 h. GAPDH was used as loading control protein (*n* = 4). Significant differences were determined by ANOVA test; between groups: ^∗^*p* < 0.05; within group: ^#^*p* < 0.05.

For the first approach, ESCs were cultured in BM, in a medium supplemented with 100 μM Zn^2+^ or LIF and incubated for 2 days with LY-294002. ESCs were then cultured 5 more days prior to measurement of the pluripotency expression markers (POU5F1 and AP). After 24 h of treatment with LY-294002, AKT phosphorylation levels were reduced in all conditions, regardless of the presence of Zn^2+^ or LIF in the culture medium (Figure 2A). After 7 days of culture and PI3K/AKT inhibition, POU5F1 and AP levels dropped significantly in ESCs cultured with Zn^2+^ and in BM conditions, while their levels remained unaltered in the LIF-supplemented cells, presenting similar values to the control without LY-294002 inhibitor (Figures 2C,D). These results demonstrate that AKT signaling is involved in maintaining ESC self-renewal by Zn^2+^ but not by LIF.

In the second approach, we treated ESCs with RNAi against ZIP7 transporter. After 3 days of ZIP7 silencing, ESCs were cultured in BM and a medium supplemented with 100 μM Zn^2+^ or LIF. Figure 2B shows the western blot experiments of ZIP7 expression after silencing by RNAi. ZIP7 expression was found to drop significantly more in all conditions than with the RNAi negative control (NC).

AKT phosphorylation after ZIP7 silencing was only significantly reduced in ESCs supplemented with Zn^2+^ (Figure 2B), suggesting that zinc transport inhibition greatly affects AKT phosphorylation. Note that both ZIP7 expression and pAKT/AKT ratio followed a similar trend in the control conditions; the highest levels were found in cells supplemented with 100 μM Zn^2+^, reinforcing the hypothesis that ZIP7 activation promotes AKT phosphorylation.

We further analyzed the expression of POU5F1 and AP markers after knocking down ZIP7. In Figure 2A significant reduction can be seen in the self-renewal effect induced by Zn^2+^, which reduced the expression of POU5F1 and AP to the BM levels. It is interesting to note that ZIP7 knock down is transient, and after 6 days of culture both, the ZIP7 expression and pAKT/AKT ratio fully recovered (Supplementary Figure 2). However, silencing ZIP7 impairs the maintenance of stemness markers, even after 7 days of culture and in the presence of Zn^2+^, showing that the effect of zinc on self-renewal maintenance is dependent of the Zn^2+^ transporter ZIP7.

We then evaluated GSK3β phosphorylation to determine whether Zn^2+^-mediated AKT activation further stimulated downstream phosphorylation of AKT target substrates. ESCs were cultured in BM and a medium supplemented with 100 μM Zn^2+^or LIF. In Figure 2E no differences can be seen in the pAKT/AKT or pGSK3β/GSK3β ratios after 4 h of culture in the different conditions used. However, after 24 h both AKT and GSK3β phosphorylation increased in the Zn^2+^- supplemented cells, whilst the LIF and BM conditions showed similar lower values. These results further support the hypothesis that zinc maintains ESC self-renewal by inducing AKT phosphorylation, which then promotes phosphorylation of downstream target proteins such as GSK3β, neutralizing their activity and promoting stemness maintenance.

### Zn^2+^–Mediated AKT Activation Is Closely Associated With Intra-Nuclear Presence of Transcription Factor POU5F1

**FIGURE 3:**
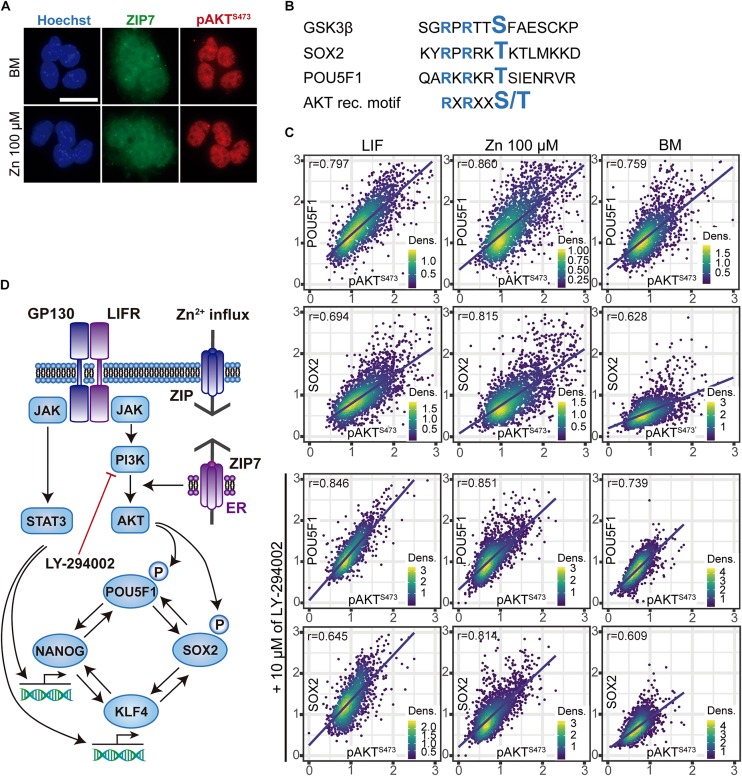
Relationship between nuclear pAKT and the core transcriptional network for ESC self-renewal. **(A)** Immunostaining of Zn^2+^ transporter ZIP7 and pAKT (S473) in ESCs cultured for 24 h in BM only or BM supplemented with 100 μM of Zn^2+^. Scale bar: 50 μm. **(B)** Alignments of aminoacid sequences of mouse GSK3β, POU5F1, and SOX2 showing the target motifs for AKT-mediated phosphorilation. NetPhos 3.1 Server was used to identify these sequences, which then were aligned with the minimal recognition motif of AKT. **(C)** Immunofluorescence detection and quantification of pluripotency markers POU5F1, SOX2, and pAKT (S473) in ESCs cultured for 3 days in normal medium and medium supplemented with 10 μM of PI3K inhibitor LY-294002. Obtained data were represented and Pearson’s correlation *r*-values were obtained using R. **(D)** Proposed mechanism illustrating the effects of Zn^2+^ in AKT phosphorylation and ESCs self-renewal. Addition of extracellular Zn^2+^ in the culture medium, activate ZIP7 and AKT downstream pathways. Note that these results have been determined experimentally in this work and the illustrated steps activated by AKT to maintain ESCs pluripotency have been previously reported in the literature (Saxe et al., 2009; Jeong et al., 2010; Lin et al., 2012).

To determine how AKT interacts with the key POU5F1 and SOX2 pluripotency regulators, we studied their intracellular location. Immunostaining for POU5F1, SOX2, and pAKT (S473) showed that they are found in the cell nuclei, whether or not exogenous Zn^2+^ is added. We also observed that cell nuclei were surrounded by the Zn^2+^ transporter ZIP7, which is anchored to the endoplasmic reticulum (Figure 3A). Like GSK3β, both SOX2 and POU5F1 possess the RXRXXS/T AKT recognition motif (Manning and Cantley, 2007; Figure 3B and Supplementary Figure 3), proteins which have been reported to be substrates for AKT phosphorylation activity (Doble, 2003; Jeong et al., 2010; Lin et al., 2012). Phosphorylation of both POU5F1 and SOX2 is critical to complete the pluripotency-associated gene expression programme (Saxe et al., 2009; Jeong et al., 2010; Lin et al., 2012). To determine whether the nuclear presence of POU5F1 and SOX2 depends on nuclear pAKT, ESCs were cultured for 3 days in BM and a medium supplemented with either LIF or 100 μM Zn^2+^. In addition, we inhibited AKT phosphorylation by adding 10 μM LY-294002 in the same experimental conditions and then analyzed the nuclear expression of POU5F1, SOX2, and pAKT by immunostaining (Figure 3C). Zn^2+^ treated cells displayed the highest correlation values between the nuclear presence of POU5F1 and SOX2 transcription factors with pAKT. Adding PI3K inhibitor produced a negative effect in pAKT, which in the cells grown in BM and Zn^2+^ was also transferred to the POU5F1 levels, while the SOX2 levels only varied slightly. These results suggest that alterations in AKT signaling strongly affect POU5F1 nuclear presence, in the absence of any additional stimuli such as STAT3 activation via LIF.

The mechanism that emerges from these experiments is represented in Figure 3D and explains how Zn^2+^ interacts with AKT and the core transcriptional network that regulates ESC pluripotency.

### Zn^2+^ Maintains ESC Pluripotency in Long-Term Cultures

**FIGURE 4:**
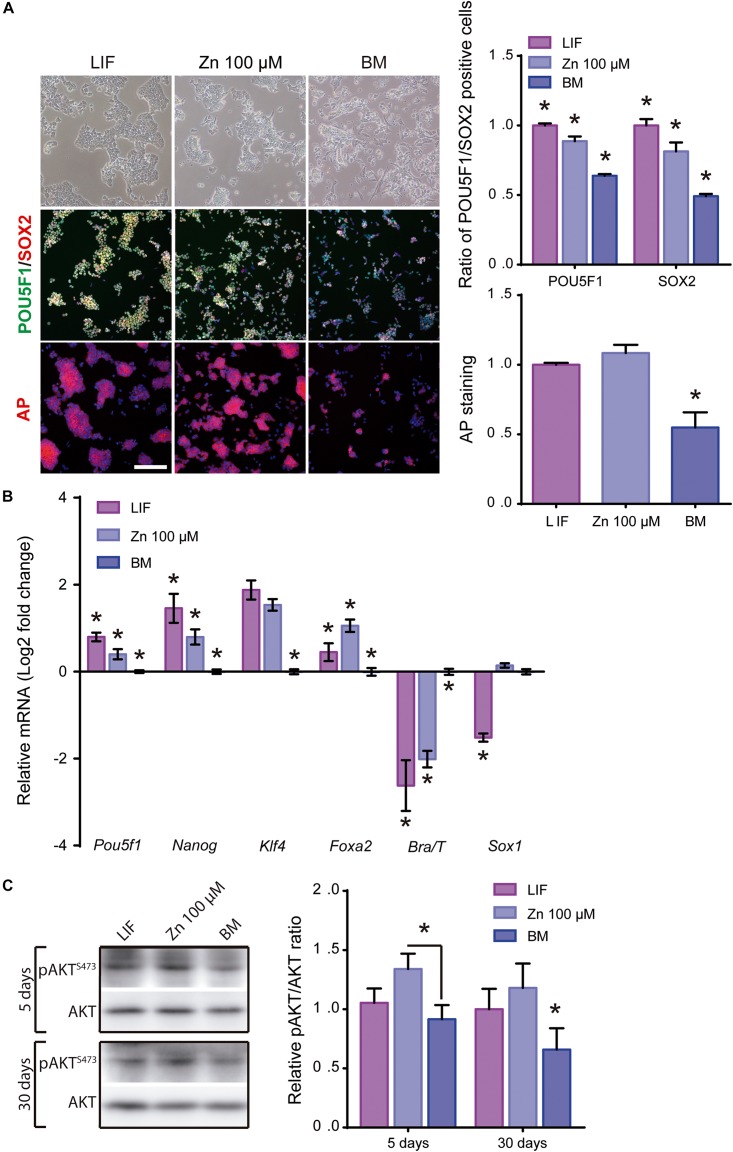
Role of zinc in ESCs pluripotency maintenance after 30 days of culture. **(A)** Immunofluorescence detection of pluripotency markers: POU5F1-green, SOX2-red and alkaline phosphatase-red, determined after 30 days of ESCs culture, in BM and a medium supplemented with LIF or medium supplemented with 100 μM of Zn^2+^ (*n* = 5). Scale bar: 200 μm. **(B)** qPCR detection of pluripotency markers (*Pou5f1*, *Nanog*, *Klf4*) and differentiation markers (endoderm: *Foxa2*; mesoderm: *Brachyury/T* and ectoderm: *Sox1*). *Gapdh* was used as housekeeping gene (*n* = 4). **(C)** Western blot detection of AKT and pAKT (S473) on ESCs cultured in BM and a medium supplemented with LIF or medium supplemented with 100 μM of Zn^2+^after 1, 5, and 30 days of culture (*n* = 4). Significant differences were determined by ANOVA test; ^∗^*p* < 0.05.

After showing that Zn^2+^ promotes ESC stemness maintenance, we then studied whether ESCs maintain pluripotency after long-term cultures in a medium supplemented with Zn^2+^. Cells were cultured in expansion conditions (seeding density: 30,000 cells/cm^2^, passaged every 2–3 days at sub-confluence) in order to reduce spontaneous differentiation (Tremml et al., 2008). After 30 days of culture in BM, LIF and 100 μM Zn^2+^, pluripotency and differentiation markers were analyzed by immunofluorescence and qPCR (Figures 4A,B). Immunofluorescence showed high POU5F1 and SOX2 expressions after 30 days of culture in the presence of Zn^2+^, with values similar to LIF and higher than the BM control (Figure 4A). Indeed, the ratio of AP positive colonies was similar for Zn^2+^ and LIF-treated cells and approximately 50% higher than for the BM condition.

Gene expression analysis of pluripotency (*Pou5f1, Nanog, KIf4*) markers showed that Zn^2+^ provided similar expression levels to LIF (Figure 4B), confirming the immunofluorescence images (Figure 4A). In contrast, for specific genes related to differentiation, *Foxa2* expression was higher for Zn^2+^ than LIF-treated cells. *Brachyury/T* expression was higher for BM cultured cells than those treated with Zn^2+^ or LIF, which displayed similar values, while *Sox1* expression was similar for Zn^2+^ and BM cultured cells (Figure 4B).

We further evaluated AKT activity after culturing ESCs for 5 and 30 days in the different conditions. Figure 4C shows that the pAKT/AKT ratio remained higher for ESCs supplemented with Zn^2+^ further supporting the hypothesis that long-term Zn^2+^-mediated stemness is mediated by AKT phosphorylation.

To disregard the aberrant chromosome content in ESCs due to long-term culture times, we analyzed ESCs karyotype after 30 days of culture (Supplementary Figure 5). No differences were found between the different conditions and all showed a modal distribution of 40 chromosomes.

### Zn^2+^ Maintains ESC Differentiation Capacity After Long-Term Cultures

**FIGURE 5:**
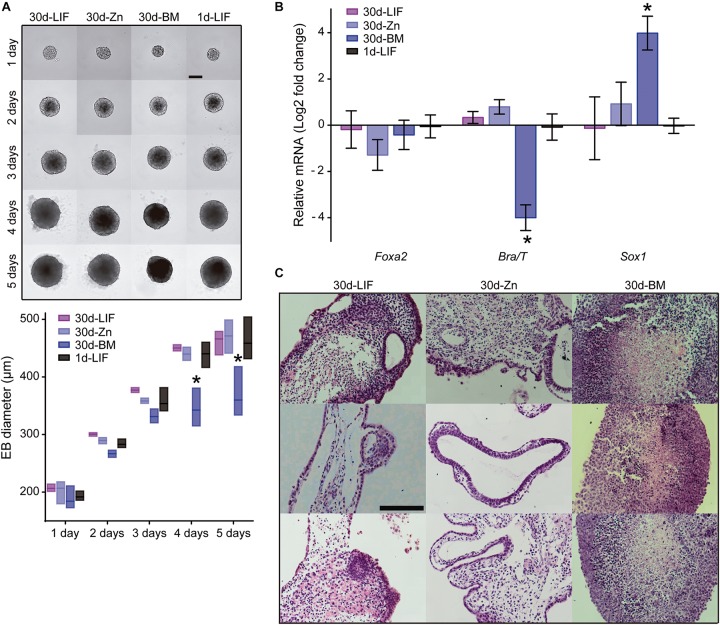
Role of zinc in spontaneous embryoid body differentiation after 30 days of culture. **(A)** Embryoid body formation of ESCs cultured for 30 days in BM and a medium supplemented with LIF or 100 μM Zn^2+^. New EBs formed from low passage ESCs (not previously cultured for 30 days under different conditions) were included as a control of embryoid body formation (1d-LIF). Measurements of EB diameters (*n* = 9). Scale bar: 200 μm. **(B)** Evaluation of spontaneous differentiation of EBs formed after culture of ESCs for 30 days in BM and a medium supplemented with LIF or 100 μM Zn^2+^. qPCR detection of primary germ layer markers: endoderm (*Foxa2*), mesoderm (*Brachyury/T*) and ectoderm (*Sox1*). *Gapdh* was used as housekeeping gene (*n* = 4). **(C)** Hematoxylin/eosin staining of histological sections of EBs after 15 days of culture. EBs were formed after culture of ESCs for 30 days in BM, medium supplemented with LIF or 100 μM Zn^2+^. Scale bar: 100 μm. Significant differences were determined by ANOVA test: ^∗^*p* < 0.05.

Having shown that Zn^2+^ maintains ESC self-renewal potential rather than differentiating into specific primary germ layers, we next evaluated whether ESC differentiation potential into different lineages was maintained after long-term cultures. ESC spontaneous differentiation capacity after 30 days of culture (30d-ESC) in BM (30d-BM), medium supplemented with 100 μM Zn^2+^ (30d-Zn) or LIF (30d-LIF) was assessed after embryoid body (EBs) formation. EBs from low passage ESCs were included as a control (1d-LIF). EBs were formed in hanging drops for 5 days and subsequently transferred to non-adhesive plates for 10 additional days. Five days old EBs were collected to analyze gene expression of primary germ layer markers. Fifteen days old EBs were collected and fixed for histological sections. No appreciable morphological differences were found between EBs obtained from 30d-Zn or 30d-LIF EBs and control EBs (1d-LIF) (Figure 5A). Nevertheless, EBs generated from 30d-BM cells had smaller diameters, which suggests less growth from the third day of culture for the different times analyzed. Gene expression related to primary germ layers rose significantly in *Sox1* (ectoderm) levels and the *Brachyury/T* (mesoderm) levels dropped sharply (Figure 5B).

We also examined the differentiation capacity of ESCs previously cultured for 30 days in BM and a medium supplemented with LIF or 100 μM Zn^2+^ under defined media conditions (Supplementary Figure 6). Similar results were found for all the induced lineages using 30d-LIF cells and 1d-LIF cells, although the 30d-Zn cells were quite different to the other conditions. For mesoderm differentiation, 30d-Zn presented lower cell density and a substantially reduced expression of CD31 and CD34 markers, together with neural-like morphology (Supplementary Figure 6B). For endoderm differentiation, 30d-Zn cells expressed SOX17, but appeared more clustered than 30d-LIF and 1d-LIF cells. Finally, for neuroectoderm differentiation, similar SOX10 and βIII-TUB images were obtained in all conditions, with 30d-BM cells showing the highest levels.

Overall, these results indicated that, as in the case of LIF-treated cells, addition of zinc to the culture medium maintains the long-term ESCs’ ability to differentiate into the three lineages.

### Zn^2+^ Maintains a Stable ESCs Pluripotent State After Long-Term Cultures

**FIGURE 6:**
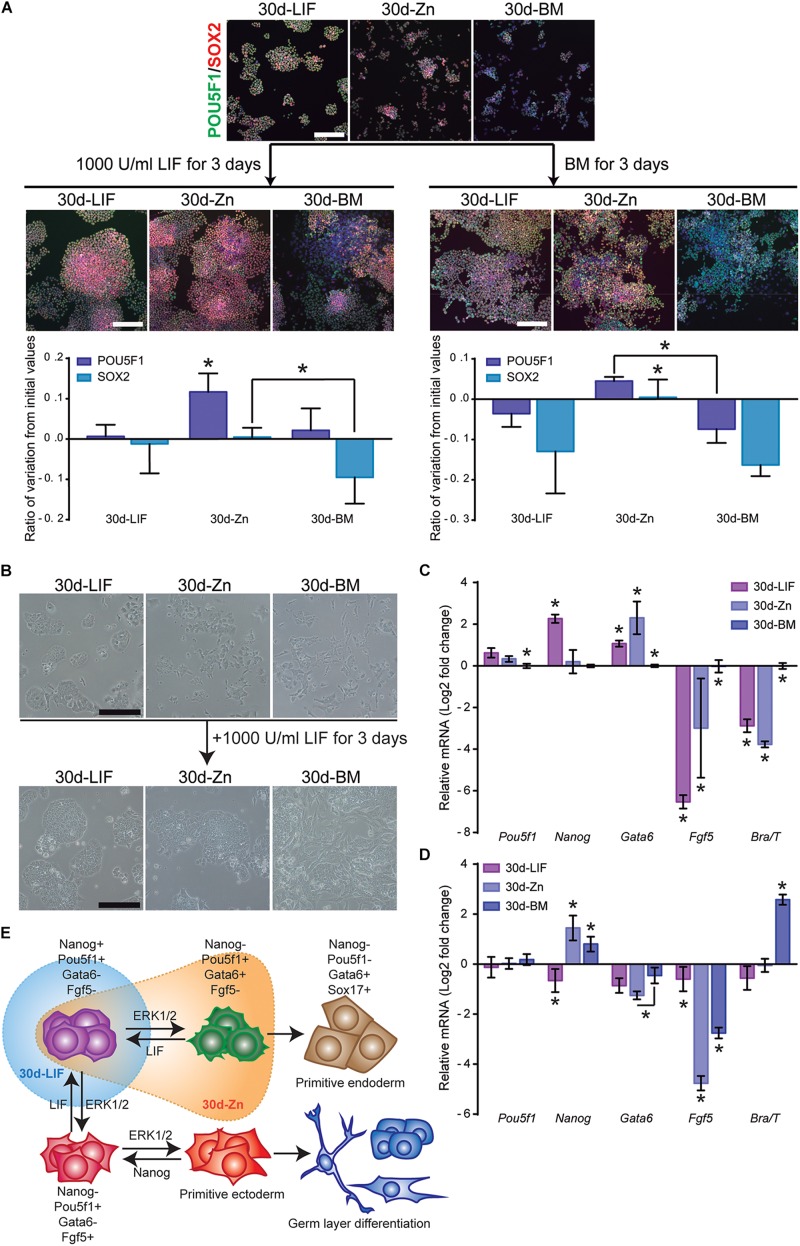
Zinc maintains naïve ESC state after long-term cultures. **(A)** ESCs previously cultured for 30 days in BM (30d-BM) and a medium supplemented with LIF (30d-LIF) or 100 μM Zn^2+^ (30d-Zn) were treated with LIF or BM for 3 more days. Pluripotency markers (POU5F1-green and SOX2-red) were determined by immunofluorescence (*n* = 5). Scale bar: 200 μm. **(B)** Bright field pictures of 30d-ESC cultured in BM (30d-BM) and a medium supplemented with LIF (30d-LIF) or 100 μM Zn^2+^ (30d-Zn) after 3 days of culture in the presence of LIF. Scale bar: 200 μm. **(C)** qPCR evaluation of stemness-related markers of pluripotency (*Pou5f1, Nanog*) indicative of naïve state, and differentiation markers (*Gata6*, *Brachyury/T*, *Fgf5*) indicative of primed state, of ESCs previously cultured for 30 days in BM (30d-BM), medium supplemented with LIF (30d-LIF) or 100 μM Zn^2+^(30d-Zn). *Gapdh* was used as housekeeping gene (*n* = 4). **(D)** qPCR evaluation of stemness related markers of pluripotency (*Pou5f1, Nanog*) indicative of naïve state, and differentiation markers (*Gata6*, *Brachyury/T*, *Fgf5*) indicative of primed state, of ESCs previously cultured for 30 days in BM (30d-BM), medium supplemented with LIF (30d-LIF) or 100 μM Zn^2+^ (30d-Zn) and treated with LIF for 3 more days in order to reverse primed to naïve state. *Gapdh* was used as housekeeping gene (*n* = 4). **(E)** Scheme of ESC pluripotency dynamics, indicating the state of the ESC population cultured with LIF (blue) and Zn (orange) after 30 days of culture. Significant differences were determined by ANOVA test; ^∗^*p* < 0.05.

As the ESC population is highly heterogeneous (Nair et al., 2015), it contains naïve cells together with other cells primed for differentiation states, with transcriptome variations concerning the pluripotency associated gene expression (Lanner and Rossant, 2010). Our results show that 30d-Zn cells maintained their self-renewal capacity and also spontaneous differentiation toward all primary germ layers in EBs, but with lower levels of mesoderm-directed differentiation (Supplementary Figure 5B). An additional experiment was carried out to determine whether this behavior is related to ESC heterogeneity after long-term cultures. ESCs previously cultured for 30 days in BM (30d-BM) and a medium supplemented with LIF (30d-LIF) or 100 μM Zn^+2^ (30d-Zn) were then treated with LIF for 3 more days in an attempt to revert ESC priming to the naïve state. Figure 6 shows immunofluorescence and qPCR results for pluripotency (*Pou5f1*, *Nanog*, SOX2) and differentiation (*Gata6, Fgf5, Brachyury*) markers. When 30d-Zn cells were exposed to LIF, the ratio of POU5F1 positive cells increased by ca. 12%, while 30d-LIF and 30d-BM levels remained constant. SOX2 values decreased for 30d-BM cells, whilst 30d-Zn and 30d-LIF cells levels did not vary significantly. In parallel, ESCs previously cultured for 30 days in BM (30d-BM) and a medium supplemented with LIF (30d-LIF) or 100 μM Zn^2+^ (30d-Zn) were treated for 3 days in BM as a control for LIF-mediated signaling. We found that 30d-LIF and 30d-BM conditions reduced the POU5F1 and SOX2 levels from the initial values, whilst the 30d-Zn levels remained almost the same (Figure 6A).

Before the 3-day LIF treatment (Figure 6C), similar results were obtained after gene expression analysis. *Nanog* expression was significantly higher for 30d-LIF cells, while 30d-Zn and 30d-BM cells showed a similar expression. Furthermore, *Gata6* was more expressed in 30d-LIF and 30d-Zn cells, while other differentiation markers (*Fgf5*, *Brachyury*) appeared to be less than the 30d-BM cells. After 3 days of LIF treatment (Figure 6D), the 30d-Zn cells rescued *Nanog* expression, with similar values to 30d-LIF cells, and the expression of *Gata6* and *Fgf5* differentiation markers decreased substantially, suggesting reversion from the primed to naïve state. Although 30d-BM cells exhibited a similar trend for *Nanog*, *Gata6* and *Fgf5* levels, after 3 days of LIF treatment the differences were significantly higher in the 30d-Zn cells. Note that *Brachyury* expression increased substantially for 30d-BM cells despite the LIF treatment (Figure 6D), suggesting incomplete transition from primed to naïve state for this condition.

### The Combination of LIF and Zn^2+^ Work Synergistically to Maintain Pluripotency

**FIGURE 7:**
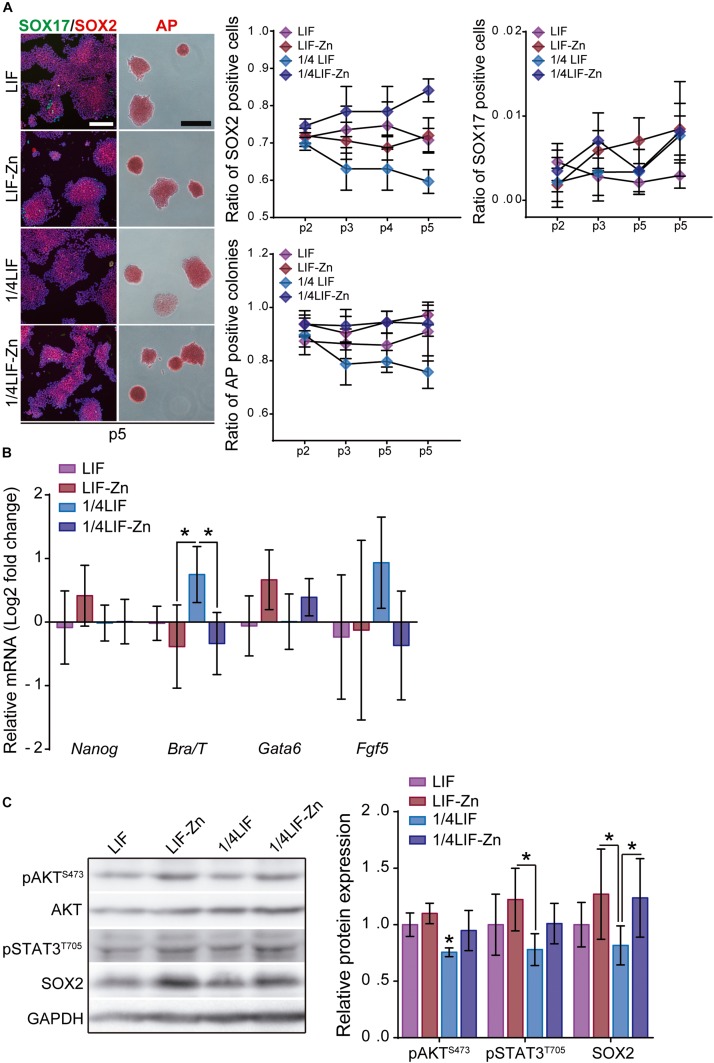
The combination of LIF with Zn^2+^ sustains ESC pluripotency with greater efficiency. **(A)** ESCs were cultured for 15 days, passaging cells each 3 days, in presence of either 1,000 U/ml of LIF (LIF), 1,000 U/ml LIF + 100 μM Zn^2+^ (LIF-Zn), 250 U/ml of LIF (1/4 LIF), and 250 U/ml LIF + 100 μM Zn^2+^ (1/4LIF-Zn). After each passage, ESCs were fixed and stained for alkaline phosphatase (AP) (*n* = 4) and immunostained for SOX2 and SOX17 (*n* = 5). Scale bar: 200 μm. **(B)** qPCR evaluation of stemness-related markers of pluripotency (*Nanog*) indicative of naïve state, and differentiation markers (*Gata6*, *Brachyury/T*, *Fgf5*) indicative of primed state, of ESCs previously cultured for 15 days. *Gapdh* was used as housekeeping gene (*n* = 4). **(C)** Western blot detection of AKT, pAKT (S473), pSTAT3 (T705), and SOX2 on ESCs cultured for 15 days. GAPDH was used as a housekeeping gene (*n* = 4). Significant differences were determined by ANOVA test; ^∗^*p* < 0.05.

Our results show that Zn^2+^ successfully sustains ESC pluripotency. Nevertheless, although 30d-Zn ESCs share many features with 30d-LIF ESCs, these cells possess particular traits, which suggest that 30d-Zn cells are more differentiated. As previous observations had indicated that the combination of LIF and Zn^2+^ strongly inhibited ESC differentiation (Figure 1D), we therefore carried out additional experiments combining different concentrations of LIF (LIF: 1,000 U/ml and 1/4LIF: 250 U/ml) and Zn^2+^ (100 μM) to enhance pluripotency maintenance. ESCs were cultured for 15 days (5 passages) in the presence of LIF, 1/4LIF, LIF-Zn, and 1/4LIF-Zn. In each passage, cells were fixed and the ratio of AP positive colonies determined as well as SOX2 and SOX17 positive cells. After 15 days, the expression of both pluripotency (*Nanog*) and differentiation (*Brachyury/T*, *Gata6*, and *Fgf5*) markers was analyzed by qPCR and protein expression was analyzed by Western Blot. Results indicate that ESCs grown with 1/4LIF gradually lost pluripotency after 15 days (Figure 7A). These cells had lower ratios of AP positive colonies and SOX2 positive cells in addition to higher *Brachyury* and *Fgf5* expressions (Figure 7B). In addition, cells cultured with 1/4LIF also had the lowest AKT activity and STAT3 phosphorylation. Interestingly, adding 100 μM of Zn^2+^ to this condition (1/4LIF-Zn), reinforced pluripotency to levels comparable to ESCs cultured with 1,000 U/ml LIF (LIF) (Figure 7C). On the other hand, the combination of LIF with 100 μM Zn^2+^ led to a higher *Nanog* expression than other conditions, and a significantly higher amount of pSTAT3 than ESCs in the presence of 1/4LIF only. However, the conditions that contained Zn^2+^ also expressed slightly higher *Gata6* values.

## Discussion

Although the need for zinc in biological processes has often been associated with its function as a cofactor in metalloproteins (Anzellotti and Farrell, 2008), its role in signaling processes is becoming increasingly important. Describing Zn^2+^ as a second messenger (Yamasaki et al., 2007) helps to explain the mechanisms associated with Zn^2+^ signaling as a useful tool for controlling cell fate. However, our results indicate that adding 40, 100, and 140 μM Zn^2+^ affected neither cell viability nor proliferation as compared to LIF or BM conditions (Supplementary Figure 1). Furthermore, zinc dynamics studies indicate that intracellular zinc uptake depends on the presence of extracellular zinc (Figure 1A) and is controlled by the ZIP7 transporter.

We have shown that Zn^2+^–mediated signaling promotes ESC self-renewal (Figure 1). The expression pattern of the pluripotency markers was similar after 100 μM Zn^2+^ and LIF supplementation, indicating that zinc inhibited spontaneous ESC differentiation and maintained the self-renewal potential rather than differentiating into specific germ layers, even under accelerated differentiation conditions (Figure 1D).

To better understand the underlying mechanisms involved in Zn^2+^–dependent ESCs stemness maintenance, we focused on PI3K/AKT activation as a consequence of the Zn^2+^ wave (Taylor et al., 2012). Inhibition of PI3K/AKT activity by LY-294002 significantly increased ESC differentiation marker levels in zinc and BM conditions, whereas the LIF condition remained unaltered (Figure 2). Although more subtly, similar results were obtained for Zn^2+^ treated cells after ZIP7 silencing (Figure 2). These findings are supported by previous works describing the link between ZIP7 and Zn^2+^–mediated AKT activation (Taylor et al., 2008, 2012). Our results suggest that ZIP7 plays a key role in Zn^2+^ mediated ESC self-renewal. It must be emphasized that LIF does not act exclusively via AKT activation to inhibit spontaneous ESC differentiation, as neither AKT inhibition nor ZIP7 silencing affected LIF–treated cells (Figure 2). The observed differences could be related to the mechanism whereby these molecules (Zn^2+^ or LIF) inhibit ESC differentiation. It has been reported that LIF promotes pluripotency gene expression by activating the transcription factor STAT3 and PI3K/AKT phosphorylation via JAK (Murray and Edgar, 2001; Rose-John, 2002). Instead the Zn^2+^ mechanism would be associated only with PI3K/AKT signaling, downstream activated after stimulating the ZIP7 transporter with extracellular Zn^2+^as we have shown in Figures 2, 3 (Taylor et al., 2008, 2012). Activating the protein kinase AKT entails the phosphorylation of its target substrates, such as GSK3β, which is inactivated after phosphorylation and stops promoting ESC differentiation (Fang et al., 2000; Bechard and Dalton, 2009; Figure 2E).

The role of AKT in ESC self-renewal has already been demonstrated by several authors (Watanabe et al., 2006; Yu and Cui, 2016). In addition, we have also observed that phospho-AKT (S473) as well as ZIP7 are essentially located in the ESC nuclei (Figure 3), which, in response to the increased extracellular Zn^2+^ concentration promotes AKT phosphorylation. It is likely that the AKT subcellular location makes it more available for substrates involved in ESC self-renewal in the same subcellular compartment such as POU5F1 (Lin et al., 2012) or SOX2 (Jeong et al., 2010; Supplementary Figure 3). Furthermore, we also observed that the co-localization of POU5F1 and SOX2 increased in Zn^2+^-treated ESCs (Supplementary Figure 4), supporting the hypothesis that the development of POU5F1-SOX2 heterodimers activates pluripotency related gene expression. Moreover, AKT is also a strong anti-apoptotic agent, targeting and inactivating pro-apoptotic proteins such as BAX (Yamaguchi and Wang, 2001). It was recently reported that ESCs lacking BAX and BAK are unable to exit from pluripotency (Wang et al., 2015), adding more relevance to AKT activity in ESC self-renewal.

Importantly, we have also shown that Zn^2+^ signaling is strong enough to promote ESC self-renewal even after 30 days of culture, maintaining both ESC–associated pluripotency markers and also AKT phosphorylation (Figure 4). Further, 30d-Zn treated ESCs spontaneously differentiate after EB formation, similarly to 30d-LIF and 1d-LIF conditions (Figure 5). When ESC differentiation was triggered by external stimuli, 30d-Zn cells differentiated toward all primary germ layers in a monolayer culture, notwithstanding the lack of mesoderm-directed differentiation (Supplementary Figure 6). We hypothesize that this lack of mesoderm differentiation is due to the pluripotency state of Zn^2+^ maintained ESC (Weinberger et al., 2016). As we have also observed, after treatment of 30d-Zn cells with 1,000 U/ml LIF, seeking to revert ESC priming to the naïve state by counterbalancing pro-differentiation signals (Lanner and Rossant, 2010; Weinberger et al., 2016), 30d-Zn cells rescued *Nanog* expression, whereas differentiation-associated genes expression (*Gata6*, *Fgf5*, *Brachyury/T*) dropped significantly, indicating an efficient transition from the primed to naïve state (Figure 6). Even though these 30d-Zn cells are in a more differentiated stage than LIF supplemented ESCs, their phenotype is highly reversible. The heterogeneity of the 30d-Zn cells and their being more primed for the differentiation state would explain their different response to differentiation signals than the LIF cultured cells. Furthermore, the higher expression of *Gata6* suggests a considerable population of ESCs primed for endoderm linage (Lanner and Rossant, 2010; Hamilton and Brickman, 2014).

The incorporation of Zn^2+^ has been shown to be enough to maintain ESC pluripotency. Further, the combination of Zn^2+^ with LIF showed a synergistic effect, which successfully increased ESC self-renewal, even in differentiation conditions (Figure 1D). The combination of Zn^2+^ and LIF not only prevented ESC differentiation significantly more than just LIF or Zn^2+^, but also enhanced the effect of LIF after long-term cultures (Figure 7). Although we have demonstrated that LIF and Zn^2+^ maintain pluripotency throughout different mechanisms, the combination of both reinforced AKT and STAT3 activity. This synergistic effect allows reduced concentration of LIF from 1,000 U/ml to 250 U/ml without loss of ESC self-renewal, maintaining ESCs pluripotent, without reducing the expression of pluripotency markers even after 15 days. By contrasts, the monotonically reduction in the number of AP positive colonies and SOX2 positive cells, together with the higher expression of *Brachyury*, demonstrated that just 250 U/ml of LIF is not enough to maintain ESC pluripotency.

## Conclusion

In conclusion, we found Zn^2+^ to be a small molecule capable of sustaining a stable ESC state as well as increasing the LIF effect in both expansion and differentiation conditions. The Zn^2+^-dependent activity of ESC self-renewal is mediated via ZIP7 and AKT activation and is maintained over a long time (more than 30 days) without additional growth factors or soluble molecules. Further, Zn^2+^-treated ESCs maintained pluripotency after long-term cultures and successfully differentiated to all primary germ layers. We therefore propose Zn^2+^ as a novel approach to control ESC fate regulation in cellular therapies.

## Data Availability

The datasets generated for this study are available on request to the corresponding author.

## Author Contributions

HM, MS-S, and PR contributed to conception and design of the study. HM wrote the first version of the manuscript, performed the experiments, and made figures. PR and MS-S supervised the research and wrote the final version of the manuscript. All authors contributed to manuscript revision, read and approved the submitted version.

## Conflict of Interest Statement

The authors declare that the research was conducted in the absence of any commercial or financial relationships that could be construed as a potential conflict of interest.
