# Supplementary material for: Zinc Maintains Embryonic Stem Cell Pluripotency and Multilineage Differentiation Potential via AKT Activation
Source: Front Cell Dev Biol. 2019 Aug 30;7:180. doi: 10.3389/fcell.2019.00180 (PMC6728745; doi:10.3389/fcell.2019.00180)
Supplement: Supplementary file 1 [file Table_1.docx]

Supplementary Material

**1. Supplementary materials and methods**

**1.1. Supplementary Methods.**

**1.1.1. ESCs viability and proliferation**

To analyze the cytotoxicity of zinc on ESCs, cells were cultured on gelatin-coated plates in BM supplemented with different concentrations of Zn^2+^ for 1, 4 and 7 days. Cell viability was assessed using Live/Dead Viability/Cytotoxicity Kit (ThermoFisher). BM without Zn^2+^ and BM supplemented with LIF were used as controls.

Cell proliferation was analyzed by quantifying total DNA concentration. For this, ESCs were seeded at very low density (1,000 cells/cm^2^) on gelatin-coated plates using BM supplemented with different concentrations of Zn^2+^ (40, 100 and 140 μM). Cells were then lysed after 1, 3, 5 and 7 days by Tris/Triton X100/EDTA (10 mMTris pH 8, 0.5% Triton X100, 1mM EDTA) buffer and DNA was quantified by Quant-iT PicoGreen dsDNA Assay Kit (ThermoFisher).

**1.1.2. ESCs differentiation experiment**

ESCs cultured for 30 days (30d-ESC) in BM (30d-BM), BM supplemented with Zn 100 µM (30d-Zn) or LIF (30d-LIF) and ESCs cultured for a short time in growth medium (1d-LIF) were differentiated as follows:

For neuroectoderm differentiation, ESCs were cultured for 6 days as a monolayer culture on plates previously coated with Laminin (10 µg/mL, Sigma-Aldrich) and using N2B27 differentiation medium as previously described (Ziomek et al., 1990).

For mesoderm differentiation, ESCs were cultured in suspension for 2 days in a serum-free (SF) medium (Fischer et al., 2008). After incubation, spheroids were collected and the SF medium was supplemented with differentiation factors: 10 µM Activin A, 10 µM BMP4 and 10 µM VEGF (Peprotech). After 4 days of culture under differentiation conditions, spheroids were disaggregated using 0.025% Trypsin/EDTA (Gibco) and further seeded on fibronectin coated plates (10 µg/mL, Sigma-Aldrich) to allow differentiated cells to spread for subsequent analysis. Cells were cultured in SF medium supplemented with differentiation factors for 2 additional days.

Finally, in order to induce monolayer differentiation to endoderm lineage, ESCs were seeded in basal medium on 0.2% gelatine-coated dishes. After 24 h of culture, the medium was substituted for endoderm differentiation medium, as described previously^52^, supplemented with 50 µM Activin A. Cells were cultured for 6 days in these conditions to induce differentiation.

## 1.2. Supplementary Tables

**Supplementary Table 1**: Antibodies for immunofluorescence assay

| **Primary antibodies** | | |
| --- | --- | --- |
| **Antibody** | **Dilution** | **Reference** |
| Goat anti- Pou5f1 | 1/400 | SantaCuz Biotech. |
| Rabbit anti-Sox2 | 1/500 | ThermoFisher |
| Rabbit anti-Sox10 | 1/500 | Abcam |
| Goat anti-Sox17 | 1/500 | R&D systems |
| Goat anti-Zip7 | 1/300 | SantaCuz Biotech. |
| Rabbit anti-β-catenin | 1/500 | ThermoFisher |
| Mouse anti-βIII-tubulin | 1/1000 | Neuromics |
| Rabbit anti-S100 | 1/300 | Sigma-Aldrich |
| Rabbit anti-CD31 | 1/300 | Abcam |
| Rabbit anti-CD34 | 1/300 | Abcam |
| Mouse anti-phospho Akt S473 | 1/500 | Millipore |
| **Secondary antibodies** | | |
| Alexa 488 conjugated goat anti-mouse | 1/700 | ThermoFisher |
| Alexa 555 conjugated goat anti-mouse | 1/700 | ThermoFisher |
| Alexa 488 conjugated goat anti-rabbit | 1/700 | ThermoFisher |
| Alexa 555 conjugated goat anti-rabbit | 1/700 | ThermoFisher |
| Alexa 633 conjugated goat anti-rabbit | 1/300 | ThermoFisher |
| DyLight 488 conjugate donkey anti-goat IgG | 1/700 | ThermoFisher |

**Supplementary Table 2**: Antibodies for western blot

| **Primary antibodies** | | |
| --- | --- | --- |
| **Antibody** | **Dilution** | **Reference** |
| Goat anti-Zip7 | 1/300 | SantaCruz Biotech. |
| Rabbit anti-Akt | 1/1500 | ThermoFisher |
| Mouse anti-phospho Akt (serine 473) | 1/1500 | Millipore |
| Mouse anti-phospho Stat3 (threonine 705) | 1/1500 | Millipore |
| Rabbit anti-Gsk3β | 1/1500 | ThermoFisher |
| Rabbit anti-phospho Gsk3β (serine 9) | 1/1000 | ThermoFisher |
| Rabbit anti-Gapdh | 1/3000 | ThermoFisher |
| **Secondary antibodies** | | |
| Rabbit anti-goat HRP conjugate | 1/10000 | ThermoFisher |
| Donkey anti-mouse HRP conjugate | 1/10000 | GE Healthcare |
| Donkey anti-rabbit HRP conjugate | 1/10000 | GE Healthcare |

**Supplementary Table 3**: RT-qPCR primer sequences

| **Primers** | | **Sequence** |
| --- | --- | --- |
| *Pou5f1* | 5’-Forward | ATGCCGTGAAGTTGGAGAAG |
|  | 5’-Reverse | TGTACCCCAAGGTGATCCTC |
| *Nanog* | 5’-Forward | CCTGAGCTATAAGCAGGTTAAGACC |
|  | 5’-Reverse | TGGATGCTGGGATACTCCAC |
| *Klf4* | 5’-Forward | AACATGCCCGGACTTACAAA |
|  | 5’-Reverse | TTCAAGGGAATCCTGGTCTTC |
| *Foxa2* | 5’-Forward | TAGCGGAGGCAAGAAGACC |
|  | 5’-Reverse | CTTAGGCCACCTCGCTTGT |
| *Brachyury/T* | 5’-Forward | GGTGGCTTGTTCCTGGTGC |
|  | 5’-Reverse | GTAGGTGGGCTGGCGTTAT |
| *Sox1* | 5’-Forward | AGACAGCGTGCCTTTGATTT |
|  | 5’-Reverse | TGGGATAAGACCTGGGTGAG |
| *Gata6* | 5’-Forward | ACTGAAGTAAGAAGAGATGGGCTTT |
|  | 5’-Reverse | ATCTCTCAATCTTCCTTAGCAGACA |
| *FgF5* | 5’-Forward | AAAGTCAATGGCTCCCACGAA |
|  | 5’-Reverse | GGCACTTGCATGGAGTTTTCC |
| *Gapdh* | 5’-Forward | AGGTCGGTGTGAACGGATTTG |
|  | 5’-Reverse | TGTAGACCATGTAGTTGAGGTCA |

**2. Supplementary results**

**
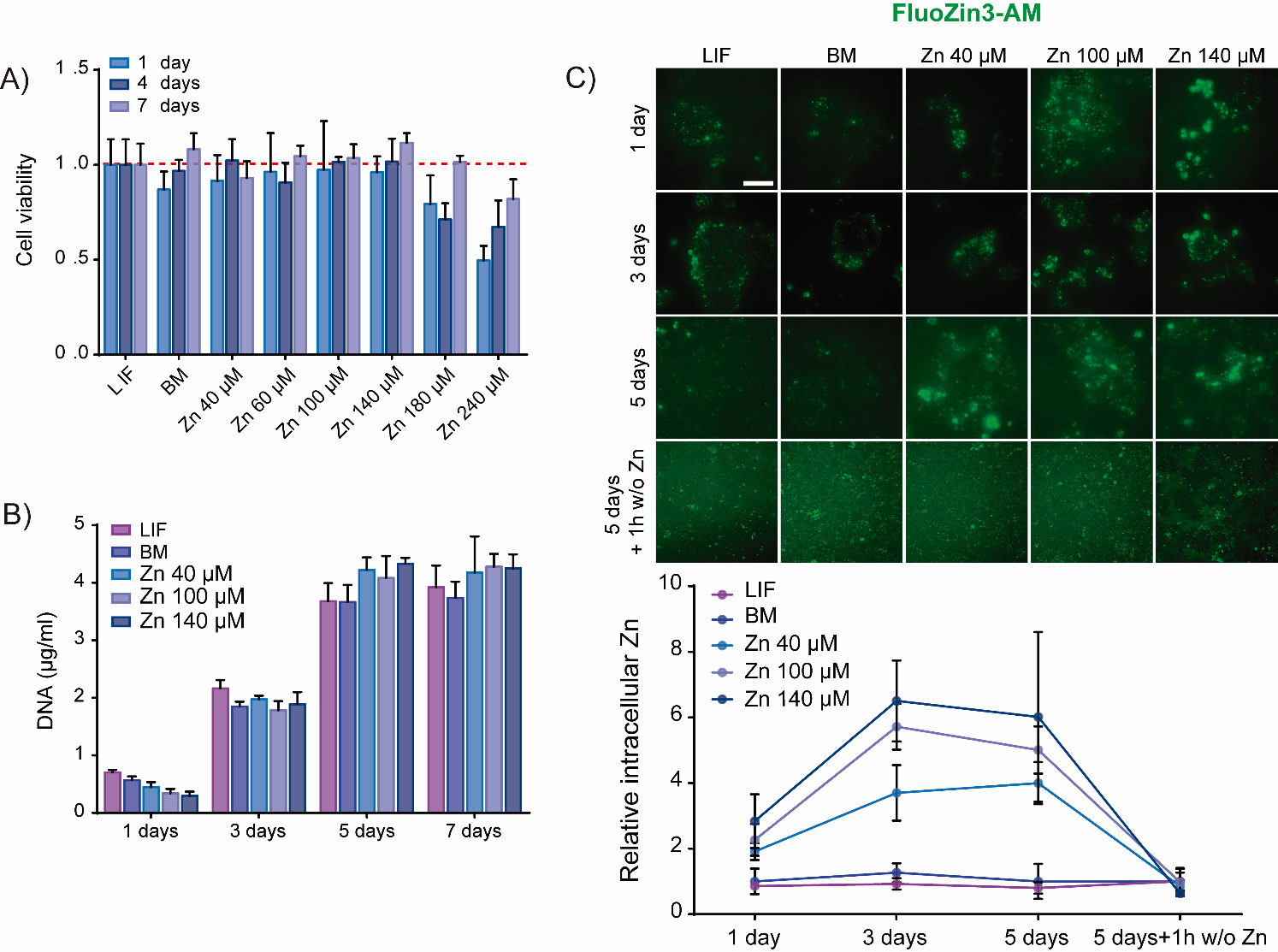
**

**Supplementary Figure 1.** Effects of zinc in ESCs viability, proliferation and intracellular Zn^2+^.

A) ESC viability after exposure to different concentrations of Zn^2+^ analyzed by live/dead assay after 1, 4 and 7d of culture. Viable cells were determined by Calcein3-AM fluorescence (n = 4). Scale bar: 200 µm.

B) Proliferation activity of ESCs cultured with different concentrations of Zn^2+^ and analyzed by total DNA quantification after 1, 3, 5, and 7 days of culture. (n = 6).

C) Intracellular concentration of Zn^2+^ determined by FluoZin 3-AM after 1, 3 and 5d treatment with different concentrations of Zn^2+^. After 5d, cell medium was replaced by a medium without zinc and intracellular Zn^2+^ was measured after 1h (5d + 1h w/o Zn).

**
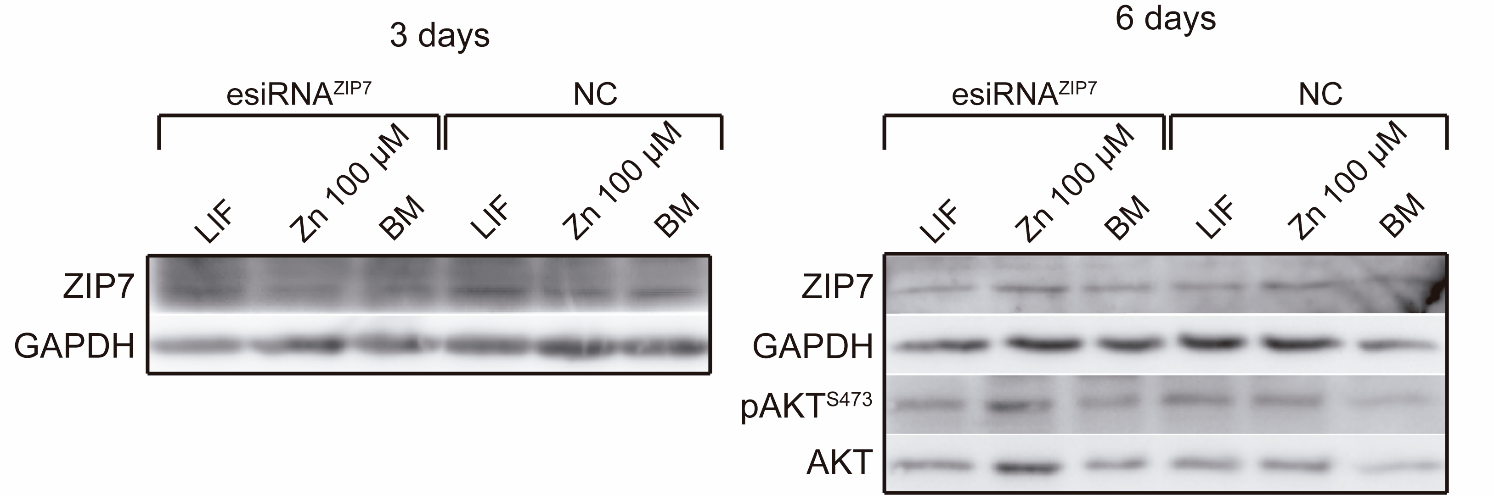
**

**Supplementary Figure 2.** Zip7 expression and AKT activity in ESCs after ZIP7 silencing with RNAi after 3 and 6 days of culture.

ESC were transfected with RNA interference (esiRNA) against Zn^2+^ transporter ZIP7. Knocked-down cells were cultured for 3 and 6 days. ZIP7 expression and AKT phosphorylation in serine 473 was determined by western blot. GAPDH was used as loading control protein.


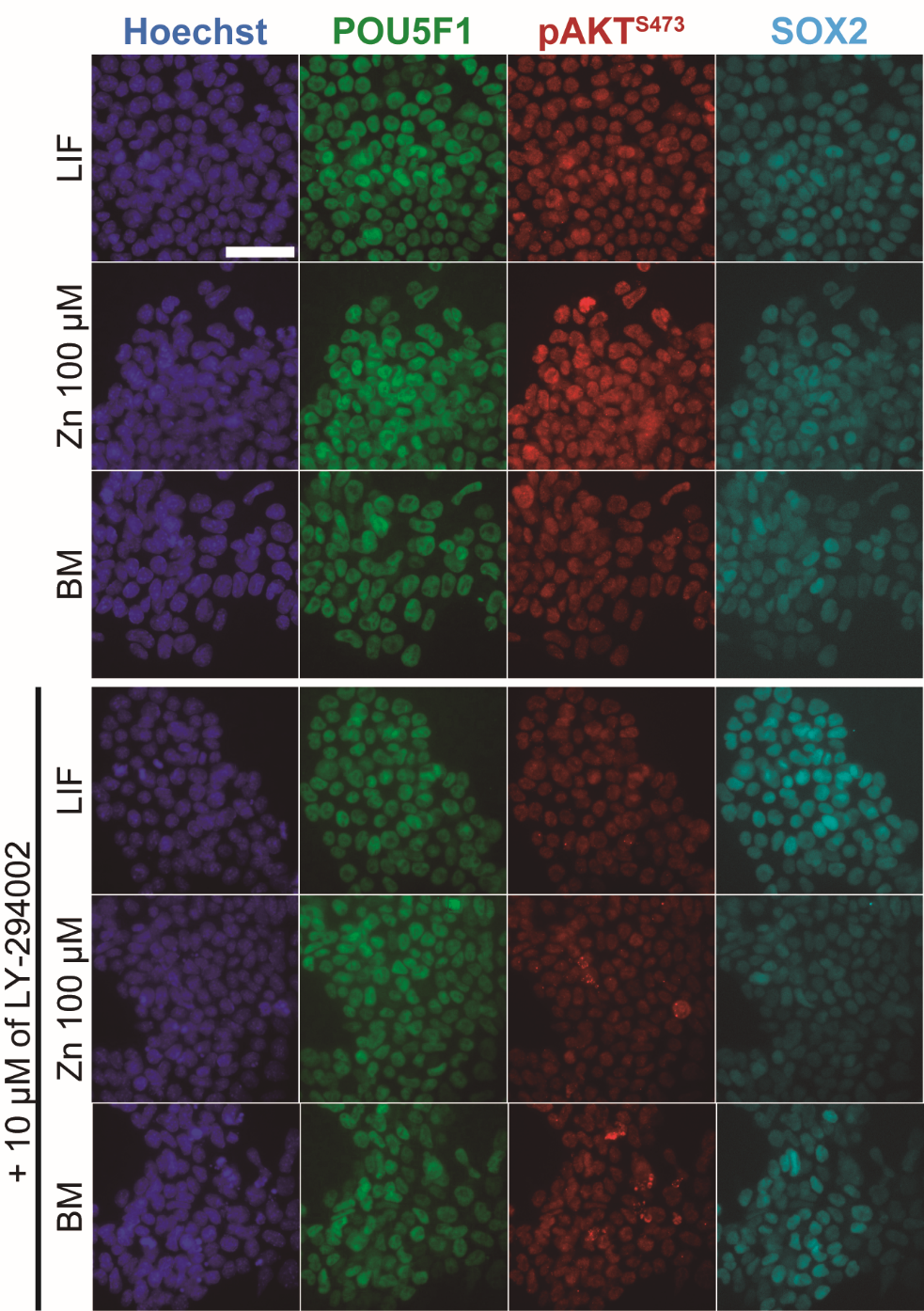


**Supplementary Figure 3.** Intracellular location of pluripotency regulators Pou5f1, Sox2 and pAkt.

Immunofluorescence images (corresponding to Figure 3) of ESCs cultured for 3 days in the different conditions and after supplementation with10 µm of PI3K inhibitor LY-294002. Scale bar: 50 µm.


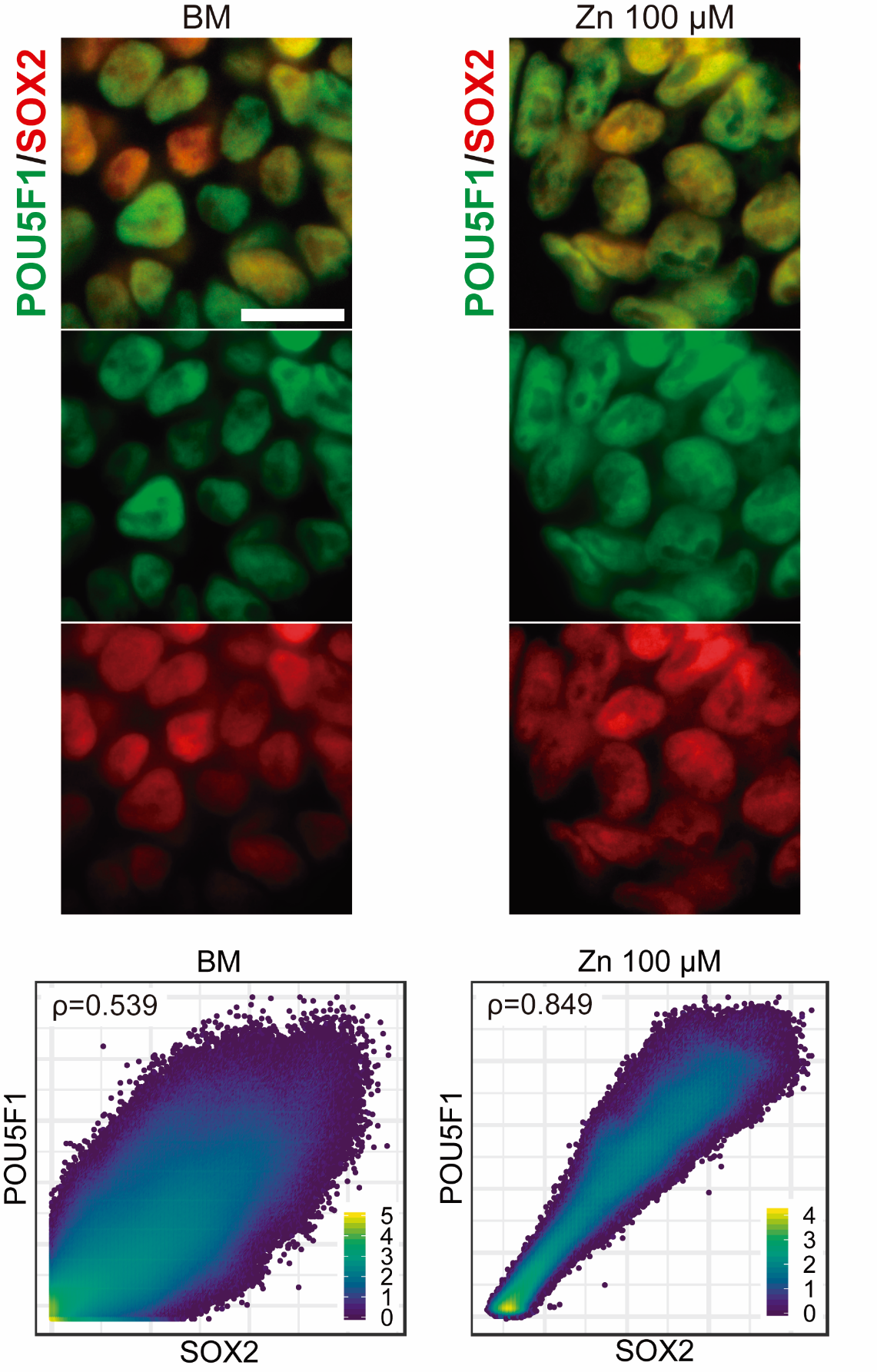


**Supplementary Figure 4.** Co-localization analysis of Pou5f1 and Sox2 in ESCs cultured for 3 days in BM and medium supplemented with 100 µM Zn^2+^.

ESCs were cultured for 3 days in BM and BM supplemented with 100 µM Zn^2+^ and the co-localization of Pou5f1 and Sox2 was assessed by immunofluorescence. Pixel correlation values between Pou5f1 and Sox2 labeling was determined by ImageJ (Coloc2). Spearman's correlation coefficient ρ was obtained for each case. Scale bar: 20 µm.

**
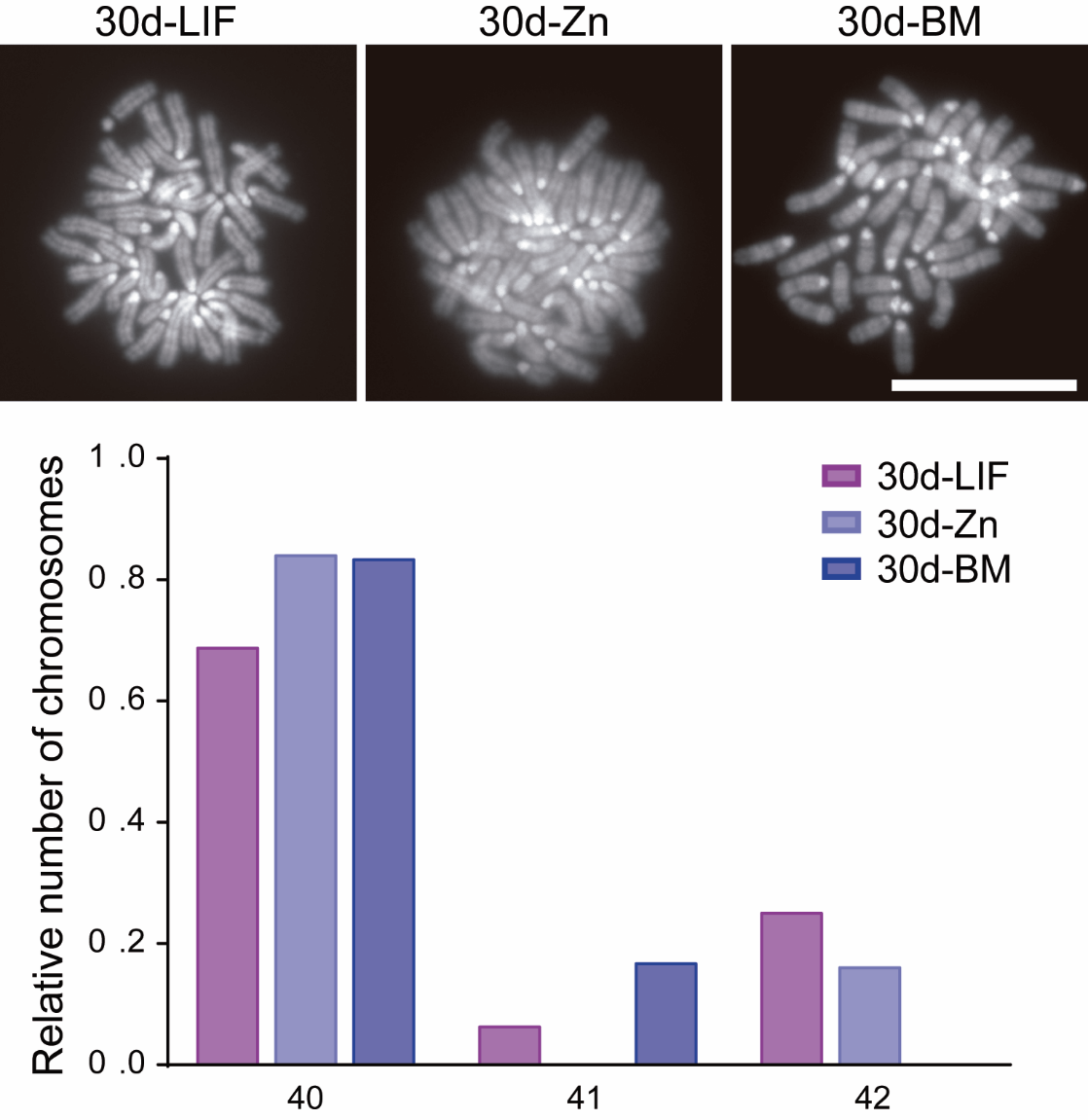
**

**Supplementary Figure 5.** ESCs karyotype after long-term culture.

Analysis of chromosome number of ESCs cultured for 30 days in BM, medium supplemented with LIF or 100 µM Zn^2+^ (n = 12). All conditions presented a modal distribution of 40 chromosomes/cell. Scale bar: 20 µm.


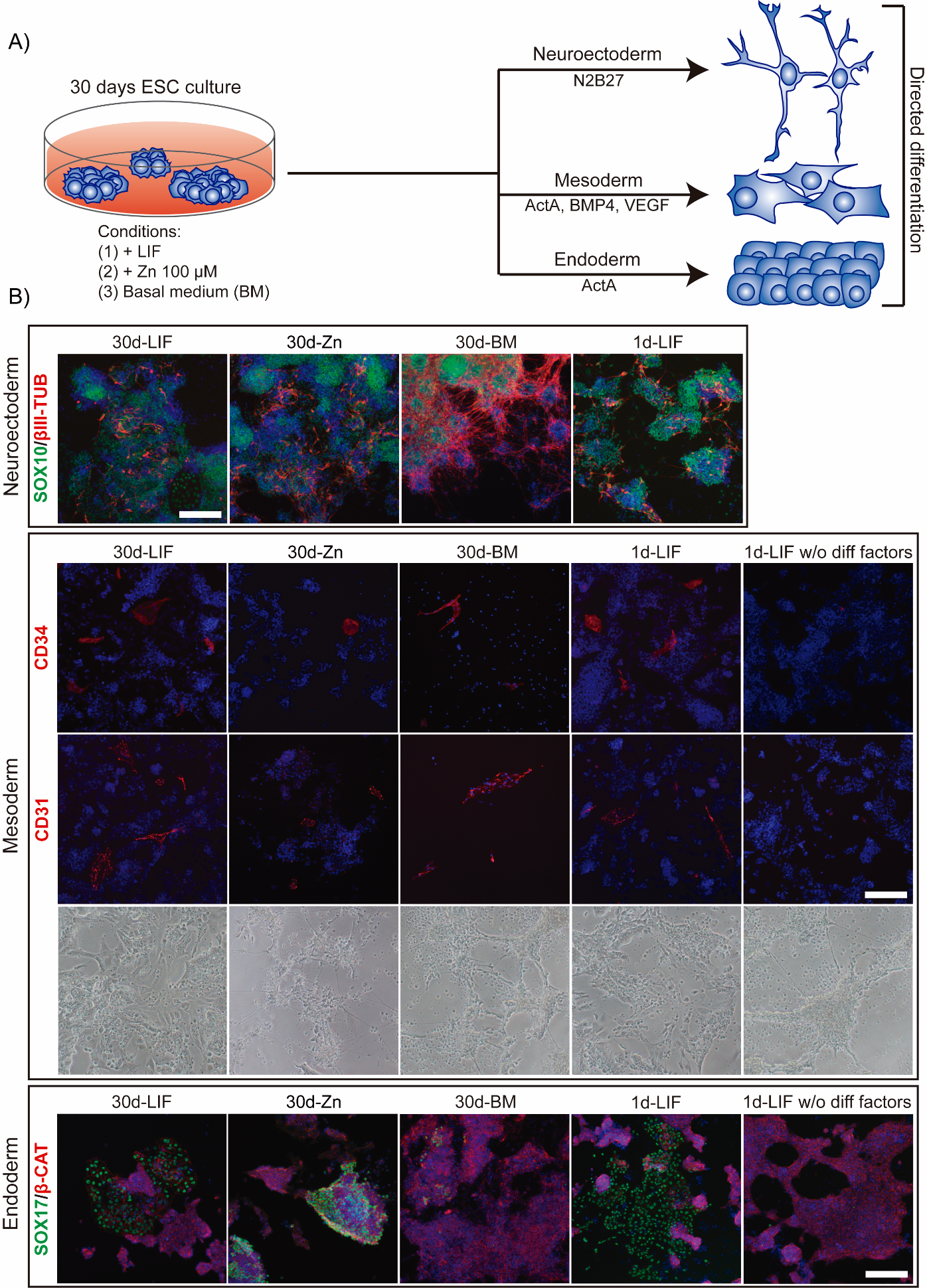


**Supplementary Figure 6.** Evaluation of differentiation ability of ESCs cultured for 30 days under different conditions.

A) Experimental design carried out to assess the pluripotent potential of ESCs cultured for 30 days in BM, medium supplemented with LIF or 100 µM Zn^2+^. After 30 days, ESC differentiation to endoderm, mesoderm or ectoderm was analyzed in EBs and in monolayer culture under defined media.

B) Analysis of differentiation potential of ESCs cultured for 30 days in BM, medium supplemented with LIF or 100 µM Zn^2+^. After 30 days culture under the different conditions, ESCs were seeded in monolayer culture with specific differentiation media. Differentiation to neuroectoderm, mesoderm or endoderm was then analyzed.

Low passage ESC with and without supplementation of differentiation factors (conditions ESC and ESC w/o diff factors) were used as a control. Differentiation markers were analyzed by immunofluoresence. Neurectoderm linage: neural linage markers βIII-tubulin (red) and Sox10 (green) were determined (n = 5). Mesoderm linage: ESCs were cultured in suspension with SF medium (markers CD31 and CD34 shown in red) (n = 5). Endoderm linage: ESCs were supplemented with Activin A (endoderm markers Sox17 (green) and β-catenin (red)) (n = 5). Scale bar: 200 µm.
